# Aggregation-induced emission from silole-based lumophores embedded in organic–inorganic hybrid hosts†

**DOI:** 10.1039/d1tc02794h

**Published:** 2021-09-16

**Authors:** Guanpeng Lyu, Thomas J. F. Southern, Bethan L. Charles, Maxime Roger, Philippe Gerbier, Sébastien Clément, Rachel C. Evans

**Affiliations:** Department of Material Science and Metallurgy, University of Cambridge 27 Charles Babbage Road Cambridge CB3 0FS UK rce26@cam.ac.uk; ICGM, Univ. Montpellier, CNRS, ENSCM Montpellier France

## Abstract

Aggregation-induced emitters – or AIEgens – are often symbolised by their photoluminescence enhancement as a result of aggregation in a poor solvent. However, for some applications, it is preferable for the AIE response to be induced in the solid-state. Here, the ability of an organic–inorganic hybrid polymer host to induce the AIE response from embedded silole-based lumophores has been explored. We have focussed on understanding how the incorporation method controls the extent of lumophore aggregation and thus the associated photophysical properties. To achieve this, two sample concentration series have been prepared, based on either the parent AIEgen 1,1-dimethyl-2,3,4,5-tetraphenylsilole (DMTPS) or the silylated analogue (DMTPS-Sil), which were physically doped or covalently grafted, respectively, to dU(600) – a member of the ureasil family of poly(oxyalkylene)/siloxane hybrids. Steady-state and time-resolved photoluminescence measurements, coupled with confocal microscopy studies, revealed that covalent grafting leads to improved dispersibility of the AIEgen, reduced scattering losses, increased photoluminescence quantum yields (up to *ca.* 40%) and improved chemical stability. Moreover, the ureasil also functions as a photoactive host that undergoes excitation energy transfer to the embedded DMTPS-Sil with an efficiency of almost 70%. This study highlights the potential for designing complex photoluminescent hybrid polymers exhibiting an ehanced AIE response for solid-state optical applications.

## Abstract 2

The incorporation of silole-based lumophores into a photoactive organic–inorganic ureasil host *via* covalent grafting or physical mixing results in enhanced photoluminescence through aggregation and host–guest energy transfer.

## Graphical abstract

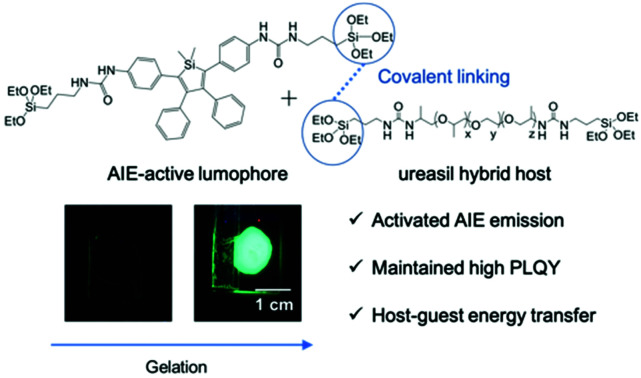

## Introduction

Organic photoluminescent materials form the basis of flexible optical devices, such as organic light-emitting diodes (OLEDs),^1,2^ organic light-emitting transistors,^3,4^ plastic lasers,^5,6^ luminescent solar concentrators (LSCs),^7–9^ and optical amplifiers.^10,11^ In these devices, the active light-emitting species, or lumophore, is often doped in a solid or liquid matrix.^3,6,11^ Poor miscibility of the lumophore with the host, particularly at high loading, can lead to aggregation of the active species, and undesirable side effects such as decreased photoluminescence efficiency (dubbed aggregation-caused quenching, or ACQ) and spectral distortion.^12^ This consequence is common for conjugated organic lumophores, for which π–π stacking interactions facilitate aggregation and switch-on non-radiative deactivation pathways.^13^ Furthermore, the presence of sufficiently large aggregates of lumophores may lead to scattering loss of the incoming photons, which can be detrimental depending on the intended application of the photoluminescent material.^14–16^ However, aggregation is not always detrimental. Some lumophores, known as aggregation-induced emitters, or AIEgens, become strongly fluorescent in the aggregated state due to the restriction of intramolecular rotations.^17,18^ Moreover, AIEgens often possess twisted-core structures which help to prevent direct π–π stacking between the aggregated molecules.^19^

The first formal report of an AIEgen was the compound pentaphenylsilole.^20^ Since then, silole-based AIEgens have been widely investigated for various applications, including fluorescent bioprobes,^21–23^ chemosensors^24,25^ and OLEDs,^25,26^ due to their good thermal and photostability, high emission efficiency, fast electron mobility and high electron affinity in the aggregated state.^25,27,28^ It is known that the photoluminescence (PL) of AIEgens can be activated not only through aggregation, but also through confinement within polymeric structures.^29^ For example, AIEgens can bind to macrocyclic hosts through non-covalent interactions in solution, forming supramolecular polymer architectures which lead to the rigidification of the AIE molecules and enhancement of their emission.^30,31^ This process can be easily reversed using external stimuli such as light and temperature, thus achieving controllable emission behaviour.^30^ The emission can also be switched-on by incorporating AIEgens into solid matrices, with the particular advantage of maintaining their photoluminescence quantum yield (PLQY) even at high concentrations.^32–35^ In addition, AIEgens have been shown to exhibit distinct emission properties depending on the microenvironment provided by the host matrix, a trait that has been exploited for visualising organic–inorganic composites,^36^ polymeric blends,^37^ and various dynamic processes such as polymerisation,^38^ gelation^39^ and self-assembly.^40^ This makes the choice of host material particularly important for tuning and optimising the optical properties of the final photoluminescent material.

In this regard, organic–inorganic hybrids prepared using sol–gel chemistry have been demonstrated as useful hosts for the dispersion of lumophores, due to the combination of the favourable properties from the organic (flexibility and easy processability) and inorganic (high stability and optical transparency) components in a single material.^41,42^ Ureasils – a family of organic–inorganic hybrids derived from poly(oxyalkylene) chains cross-linked to a siliceous framework *via* urea linkages – have shown significant potential as optical hosts due to their high refractive indices^10,11^ and excellent optical clarity.^43–45^ Ureasils themselves are intrinsically photoluminescent, capable of converting UV radiation into blue emission, with a PLQY of up to 15% depending on the synthesis conditions and the poly(oxyalkylene) used.^44,45^ Moreover, electronic coupling between the embedded lumophore may lead to enhanced PLQYs^46^ or Förster resonance energy transfer (FRET) in the presence of appropriate spectral overlap, offering the possibility for tuning the wavelength response of spectral-converting devices across a wider range of the UV-visible spectrum.^46–50^ The versatile sol–gel synthesis route offers further advantages including moderate processing temperatures to prevent decomposition of the molecular lumophore^51^ and the possibility of covalently-grafting the lumophores to the siliceous backbone of the hybrid matrix.^52,53^ This can be achieved through the use of silylated lumophores that are able to co-condense with the alkoxide precursors used to form the hybrid matrix, resulting in a cross-linked siliceous network between the lumophores and host material.^14,54,55^ Using this approach, the solubility and stability of the embedded lumophores can be enhanced, allowing for higher doping concentrations of the lumophores in the solid matrix without phase separation.^14,54,55^ In theory, by improving the dispersion of lumophores within the hybrid matrix at a given concentration, any undesirable scattering loss might also be reduced. The covalent interaction between the lumophore and the hybrid host may also help to rigidify the grafted lumophores, which further reduces non-radiative losses and improves their emission efficiencies.

**Fig. 1:**
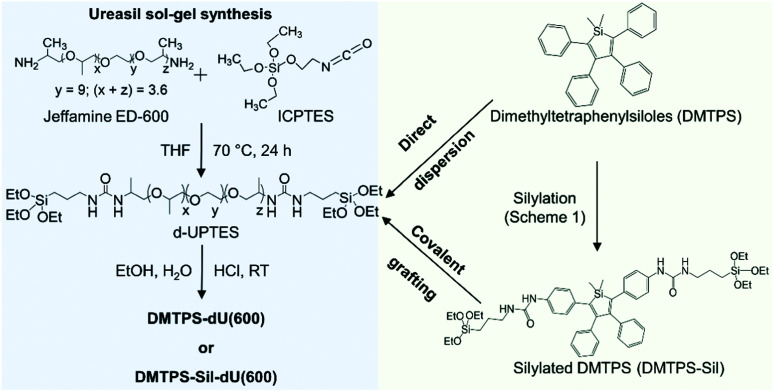
Synthesis route for the preparation of AIEgen-incorporated di-ureasils. The silole-based AIEgen, DMTPS, is directly dispersed in the di-ureasil matrix, while the silylated analogue, DMTPS-Sil, is covalently grafted to the siliceous framework due to the hydrolysis and co-condensation between the triethoxysilyl groups of DMTPS-Sil and the d-UPTES hybrid precursor under the acid-catalysed sol–gel conditions.

Herein, we investigate the ability of a di-ureasil host, dU(600), to induce aggregation, and thus emission, of silole-based AIEgens. Two methods of incorporation are investigated: (i) physical confinement of the parent lumophore, 1,1-dimethyl-2,3,4,5-tetraphenylsilole (DMTPS) and (ii) covalent grafting *via* the terminal silyl ether groups on the silylated analogue of DMTPS (DMTPS-Sil) to the siliceous backbone of the ureasil (Fig. 1). In both cases, the effect of the AIEgen concentration is also considered. Through investigation of the photoluminescence behaviour in solution, through the sol–gel transition and in final solid samples, we show that by covalently-grafting the AIEgen to the ureasil host, the AIE response is significantly enhanced. Detailed time-resolved photoluminescence studies are performed to quantify the effect of the incorporation method on the extent of aggregation of the lumophore, and also to examine the possibility of FRET from the photoactive ureasil host to the embedded lumophores.

## Experimental

### Materials

Palladium on carbon (10 wt%), bis(2-aminopropyl) polypropylene glycol-*block*-poly-ethylene glycol-*block*-polypropylene glycol (Jeffamine ED-600, *M*_w_ = 600 g mol^−1^) and 3-(triethoxysilyl)propylisocyanate (ICPTES, 95.0%) were purchased from Sigma-Aldrich. Sodium borohydride (NaBH_4_, 98%), tetrahydrofuran (THF, ≥99.9%), ethanol (EtOH, 95.0%), and hydrochloric acid (HCl, 37%) were obtained from Fisher Scientific. Dry dichloromethane (CH_2_Cl_2_) and THF were obtained using a solvent purification system, PureSolve MD5 purchased from Inert Technology (Amesbury, MA, USA). Water was obtained from a Millipore Simpak 2 water purification system (*ρ* = 18 mΩ). Lumogen F Red 305 (LR305) was a kind gift from BASF® Germany. All materials were used as received. DMTPS^56^ and 1,1-dimethyl-2,5-di(4-nitrophenyl)-3,4-diphenylsilole (DMTPS-NO_2_)^57^ were prepared according to literature procedures.

### Synthesis of DMTPS-Sil

A solution of DMTPS-NO_2_ (252 mg, 0.5 mmol) and palladium on activated carbon (10 wt%, 3 mg) in a mixture of CH_2_Cl_2_ (9 mL) and methanol (1 mL) was prepared and degassed. Then, NaBH_4_ (25 mg, 0.66 mmol) was added. The progress of the reaction was monitored by thin layer chromatography (TLC) using a CH_2_Cl_2_/*n*-hexane (1 : 1) mixture as the eluent. After completion of the reaction, the solvent was evaporated under reduced pressure. CH_2_Cl_2_ (10 mL) was added to the residue and the mixture was filtered through Celite. After evaporation of the solvent, ICPTES (1.2 mmol, 297 μL) and THF (10 mL) were added to the corresponding 1,1-dimethyl-2,5-di(4-aminophenyl)-3,4-diphenylsilole (DMTPS-NH_2_) obtained as a yellow solid. The reaction was stirred at 60 °C overnight. Then, the solvent was evaporated under reduced pressure and the residue was washed with *n*-hexane (4 × 5 mL). The solid was recrystallised in a mixture of CH_2_Cl_2_/*n*-hexane leading to DMTPS-Sil as an orange powder. Yield: 48% (230 mg). IR (ATR): 3313 cm^−1^ (νNH), 1649 cm^−1^ (*ν*CO_amide_). ^1^H NMR (300 MHz, CD_2_Cl_2_, 293 K): *δ* = 7.05–6.99 (m, 10H), 6.84–6.78 (m, 8H), 6.48 (s, 2H, N*H*CO), 5.02 (t, 2H, ^3^*J*_H–H_ = 6.3 Hz, N*H*CH_2_), 3.77 (q, 12H, ^3^*J*_H–H_ = 7 Hz, OCH_2_CH_3_), 3.16 (q, 4H, ^3^*J*_H–H_ = 6.3 Hz), 1.64–1.52 (m, 4H), 1.17 (t, 18H, ^3^*J*_H–H_ = 7 Hz, OCH_2_C*H*_3_), 0.60 (t, 4H, ^3^*J*_H–H_ = 7 Hz, CH_2_Si), 0.37 (s, 6H, SiC*H*_3_). ^13^C{^1^H} NMR (75 MHz, CD_2_Cl_2_): *δ* = 207.1, 156.0, 154.1, 141.1, 140.0, 137.4, 135.1, 130.5, 130.1, 128.0, 126.7, 120.1, 58.9, 43.2, 24.1, 18.7, 7.1, −3.3 ppm. ^29^Si{^1^H} NMR (79.6 MHz, CD_2_Cl_2_, 293 K): *δ* = 7.6, −45.6 ppm. Elemental analysis calcd for C_50_H_70_N_4_O_8_Si_3_: %C 63.93, %H 7.51; %N 5.96, found %C 64.06, %H 7.64; %N 5.98. Characterisation spectra shown in Fig. S1–S4, ESI.†

### Synthesis of AIEgen-incorporated ureasils

Di-ureasils either doped with DMTPS or grafted to DMTPS-Sil, denoted DMTPS-dU(600) and DMTPS-Sil-dU(600) respectively, were synthesised *via* a two-step sol–gel process. In the first step, ICPTES (0.91 mL, 3.68 mmol) was mixed with Jeffamine ED-600 (1.00 mL, 1.75 mmol) in THF (5 mL) under stirring, corresponding to a molar ratio of 2 : 1 between ICPTES and Jeffamine ED-600. The reaction mixture was refluxed at 70 °C for 24 h, to obtain “one batch” of the organic–inorganic hybrid precursor, diureapropyltriethoxysilane (d-UPTES) in solution. The reaction progress was monitored *via* Fourier transform infrared (FTIR) spectroscopy. The requisite volume, based on the final dopant concentrations of 0.014 mM, 0.14 mM and 1.4 mM, of stock solutions (1 mg mL^−1^) of DMTPS or DMTPS-Sil was added to the d-UPTES solution under stirring. In the second step, gelation reagents (ethanol, HCl (0.5 M) and water) were added to the d-UPTES in sequence and thoroughly mixed. The molar ratio of ICPTES : ethanol : HCl : water used was 176 : 354 : 1 : 265. The resulting mixture was poured into a polypropylene mould and gelled into free-standing monoliths. The mould was sealed with Parafilm M to allow slow evaporation of the excess THF in the samples over 5 days, followed by further oven drying at 40 °C for 3 days. “One batch” of d-UPTES was used to yield a dU(600) monolith with dimensions of 3.0 cm × 3.0 cm × 0.3 cm. Through the sol–gel process, DMTPS-Sil molecules are covalently grafted to the siliceous framework of the di-ureasil as a result of the hydrolysis and co-condensation between the triethoxysilyl functional groups of both the DMTPS-Sil and d-UPTES under the addition of the gelation reagents.^54,55^

### Dye extraction studies

DMTPS-dU(600) or DMTPS-Sil-dU(600) samples were cut into small pieces (∼2 × 2 × 2 mm each) and soaked in acetone (10 mL), a good solvent for the di-ureasil matrix, for 72 hours. The samples were then removed from the solution and dried at room temperature for 7 days. The acetone supernatant was passed through a syringe filter (0.2 μm) to remove traces of the solid samples and the emission spectra were recorded.

### NMR spectroscopy

NMR spectra were recorded either on a BRUKER AVANCE III 300 spectrometer (^1^H, 300 MHz and ^13^C{^1^H}, 75 MHz) or on a BRUKER AVANCE III 400 (^29^Si{^1^H}, 79.6 MHz) spectrometer. Chemical shifts are calibrated to trimethylsilane (TMS) based on the relative chemical shift of the residual non-deuterated solvent as an internal standard given in ppm relative to TMS and coupling constants *J* in Hz.

### Elemental analysis

Elemental analysis was performed on a Vario MICRO CHNS elemental analyzer (Elementar, LangenselboldGermany).

### Attenuated total reflectance Fourier transform infrared (ATR-FTIR) spectroscopy

ATR-FTIR transmittance spectra were recorded with a Bruker® Tensor 27 FT-IR System at room temperature. The spectra were recorded over a range of 4000–500 cm^−1^ with resolution of 4 cm^−1^ and averaged over 64 scans.

### UV/Vis absorption spectroscopy

UV/Vis absorption and transmittance spectra were measured with a PerkinElmer Lambda 750 spectrophotometer using wavelength scan with a resolution of 1 nm at a scan speed of 267 nm min^−1^ and a slit width of 2 nm. Liquid samples were analysed in a quartz cuvette with a 10 mm path length, and solid samples were directly mounted to the sample holder.

### Steady-state excitation and emission studies

Steady-state PL spectroscopy was performed on a Fluorolog-3 spectrophotometer (Horiba Jobin Yvon). Solid-state emission spectra were recorded using the front-face configuration. The excitation and emission slits were adjusted so that the maximum PL intensity was within the range of linear response of the detector and were kept the same between samples if direct comparison between the emission intensity was required. PLQYs were measured using a Quanta-phi integrating sphere (Horiba Jobin Yvon) mounted on the Fluorolog-3 spectrophotometer. The values and errors reported are the mean and standard deviation of three repeat measurements. Emission and excitation spectra were corrected for the wavelength response of the system and the intensity of the lamp profile over the excitation range, respectively, using correction factors supplied by the manufacturer.

### Confocal fluorescence microscopy (CFM)

Fluorescence images of the di-ureasil gelation process were obtained using a confocal laser scanning fluorescence microscope (Zeiss Axiovert 200M with LSM 700). A drop of d-UPTES solution in THF mixed with lumophores (DMTPS, DMTPS-Sil or LR305) and gelation reagents (EtOH, HCl and H_2_O) was cast on a microscope slide and excited with a 405 or 488 nm laser. A beam splitter was used to selectively detect the emission from the lumophores at wavelengths >520 nm. A sample image was taken every minute over a 10 minute period.

### Time-resolved emission measurements

Photoluminescence decay measurements were performed using the time-correlated single photon counting method (TCSPC) on a FLS1000 PL spectrometer (Edinburgh Instruments). Pulsed lasers of wavelengths 375 nm (EPL-375, pulse width <100 ps, pulse frequency 10 MHz) and 450 nm (EPL-450, pulse width <100 ps, pulse frequency 10 MHz) were used as excitation sources. The emission decay was recorded using a high-speed photomultiplier tube (PMT-920) equipped with TCC2 counting electronics. The full-width-half-maximum (FWHM) of the instrument response function (IRF) of the system was ∼60 ps. Reconvolution and data-fitting were performed as individual fits to each emission decay using a multiexponential decay function using the FAST software package (Edinburgh Instruments). The goodness of fit was assessed using the reduced chi-square statistics, *χ*^2^, and the randomness of the residuals.

The Förster resonance energy transfer (FRET) efficiency, *E*, can be determined by comparing the intensity decays (*I*(*t*)) of the donor in the presence and absence of the acceptor, labelled as *I*_DA_(*t*) and *I*_D_(*t*), respectively:^58^1
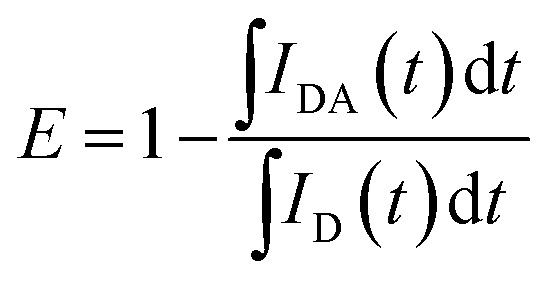


For PL emission that exhibits multi-exponential decay, *I*(*t*) is proportional to the amplitude-weighted lifetime, 〈*τ*〉, defined as:^58^2
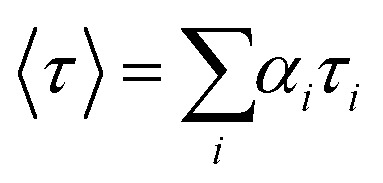
where *α*_*i*_ and *τ*_*i*_ are the normalised pre-exponential value and lifetime of each decay component, respectively. Therefore, *E* can be calculated directly from 〈*τ*〉:3
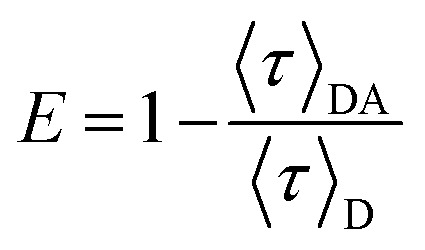
where 〈*τ*〉_DA_ and 〈*τ*〉_D_ are the amplitude-weighted lifetimes of the donor in the presence and absence of acceptor, respectively.

### Calculations

Full geometry optimization of the ground state and frequency calculations were performed by Density Functional Theory (DFT)^59^ using the hybrid Becke 3 parameters exchange functional and the Lee–Yang–Parr non-local correlation functional (B3LYP) implemented in the Gaussian 09 program suite^60^ using the 6-31G(d,p) basis set and the default convergence criterion implemented in the program. Transition diagrams were obtained through time-dependent (TD)-DFT calculations performed using the B3LYP functionals and the 6-311+G(d,p) basis set on the geometry of S_0_.

## Results and discussion

### Synthesis of the silylated silole precursor

**Scheme 1:**
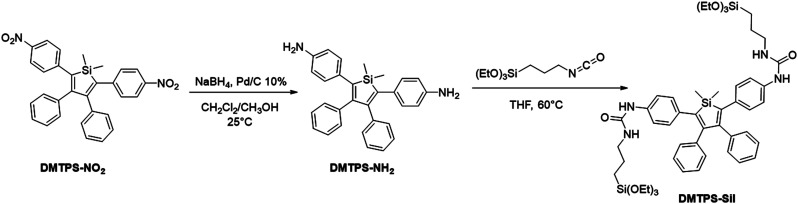
Reaction scheme for the preparation of silylated DMTPS.

DMTPS-Sil was prepared from 1,1-dimethyl-2,5-di(4-nitrophenyl)-3,4-diphenylsilole (DMTPS-NO_2_), as shown in Scheme 1. DMTPS-NO_2_ was quantitatively reduced with sodium borohydride and 10% Pd/C in a dichloromethane/methanol mixture. The corresponding 1,1-dimethyl-2,5-di(4-aminophenyl)-3,4-diphenylsilole (DMTPS-NH_2_) was not isolated but used directly for reaction with ICPTES leading to DMTPS-Sil in 48% yield. The presence of the trialkoxysilyl group was confirmed by a triplet and a quadruplet at 1.17 and 3.77 ppm in the ^1^H NMR spectrum of DMTPS-Sil, respectively assigned to the CH_3_ and OCH_2_ groups (Fig. S2 in the ESI†). As expected, the ^29^Si{^1^H} spectrum of DMTPS-Sil exhibits two signals at 7.6 and −45.6 ppm (Fig. S4 in the ESI†), corresponding to the silicon atom of the silole core and to the Si(OEt)_3_ moieties, respectively.

### Incorporation of AIEgens into the di-ureasil matrix

The di-ureasil (dU(600)) was synthesised through a two-step sol–gel process, as illustrated in Fig. 1. In the first step, the di-branched organic polymer, Jeffamine ED-600, is coupled with an alkoxysilane precursor, ICPTES to form the organic–inorganic hybrid intermediate d-UTPES in THF solution (see Fig. S5, ESI†). The name dU(600) denotes the use of the ED-600 precursor. In the second step, triggered by the addition of gelation reagents, the terminal triethoxysilyl groups on the d-UPTES are hydrolysed and co-condensed to form a siliceous backbone, resulting in a cross-linked, free-standing monolith after drying.

The dU(600) structure is typically amorphous in nature (from powder X-ray diffraction), with a relatively condensed organosilica network with a degree of condensation in the region of 70–80%.^44,46,61^ The degree of condensation is not affected by the method of incorporation, nor concentration of the organic lumophore, at least within the detection limits of solid-state ^29^Si magic-angle spinning NMR.^46^

Two methods were investigated to incorporate the AIEgen into the di-ureasil: covalent grafting and physical dispersion. In the dispersion approach, a fixed volume of DMTPS in THF is mixed with d-UPTES (also in THF) under stirring prior to the addition of the gelation reagents, which results in physical confinement of the AIEgen in the subsequently formed di-ureasil. In the grafting approach, upon addition of the gelation reagents, the terminal triethoxysilyl groups on the silylated analogue DMTPS-Sil and d-UPTES are hydrolysed and co-condensed, leading to the formation of a siliceous framework to which the DMTPS-Sil is covalently attached.^52,54,62^ Mass spectrometry studies have previously demonstrated that a silylated perylene lumophore undergoes hydrolysis and condensation under the reaction conditions used here (pH 2) and grafts to the siliceous network of the di-ureasil.^54^

The resulting solid samples are herein named DMTPS-dU(600)-*x* and DMTPS-Sil-dU(600)-*x*, respectively, where *x* refers to the concentration of AIEgen (0.014, 0.14 and 1.4 mM with respect to the final solid product). The PL behaviour of both systems will now be discussed at three stages through the synthetic process: (1) in solution; (2) as the material transitions through the sol–gel process upon addition of the gelation reagents; (3) in the final solid-state samples.

### Optical properties of AIEgens in solution

**Fig. 2:**
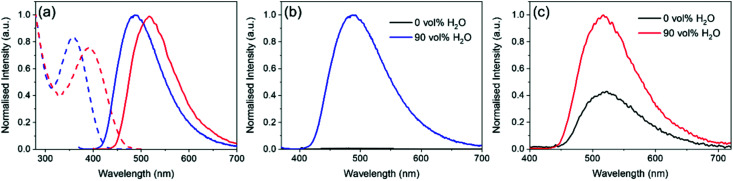
PL properties of AIEgens in solution. (a) Normalised absorption (black lines, 20 μM in THF) and emission (red lines, 20 μM in 10 : 90 THF : H_2_O volume mixture) spectra of DMTPS (solid lines) and DMTPS-Sil (dashed lines), with *λ*_ex_ (for emission spectra) = 370 and 390 nm, respectively. Emission spectra of (b) DMTPS (*λ*_ex_ = 370 nm) and (c) DMTPS-Sil (*λ*_ex_ = 390 nm) measured in pure THF (black) and 10 : 90 (v/v) THF–H_2_O mixture (red). *N.B.*: The emission intensity of DMTPS in THF (b) is negligible and lies at the baseline.

The UV/Vis absorption and PL spectra of DMTPS and DMTPS-Sil in THF (20 μM) are shown in Fig. 2a. DMTPS exhibits a broad absorption band (*λ*_abs_ = 361 nm) associated with a π–π* transition involving the HOMO (highest occupied molecular orbital) and LUMO (lowest unoccupied molecular orbital) levels (see Section S3, ESI†) and featureless green emission band (*λ*_em_ = 487 nm) associated with radiative relaxation from the S_1_ state, in good agreement with the literature.^63^ In comparison, the absorption and emission spectra of DMTPS-Sil are red-shifted, with *λ*_abs_ = 390 nm and *λ*_em_ = 517 nm, respectively. Previous studies have shown that the type and position of substituent groups can affect the electronic structure of siloles.^64^ To explore this effect, time-dependent density functional theory calculations were performed. The optimised structures (Fig. S6, ESI†) and calculations confirm that for DMPTS-Sil, the ureido groups are involved in the conjugation of the active core. This induces a strong destabilisation of the HOMO level (+0.52 eV) and stabilisation of the LUMO level (−0.24 eV) compared to the parent DMPTS, resulting in a corresponding decrease in the HOMO–LUMO gap (Fig. S7, ESI†). This result, combined with the calculated values for the absorption maxima (395 nm and 430 nm for DMPTS and DMPTS-Sil, respectively), support the observed red-shift in the experimental absorption and emission spectra of DMPTS-Sil.

Since the TD-DFT calculations have shown that the absorption maxima at 361 and 390 nm for DMTPS and DMTPS-Sil, respectively are due to S_0_ → S_1_ transitions, the calculation of the corresponding oscillator strengths, which are related to the population probability of the S_1_ state, can provide an indicator by which to estimate the PLQY.^65^ Therefore, with an oscillator strength *f* of 0.243, DMTPS should display a lower PLQY than DMTPS-Sil (*f* = 0.587). This is reflected in the moderately higher PLQY of DMTPS-Sil in solution (*Φ*_PL_ = 3.1 ± 0.1%) compared to DMTPS, which is essentially non-emissive in THF. However, interactions with the local environment (*e.g.*, solvent, neighbouring molecules) will also profoundly affect the PLQY. For example, the PL emission of AIEgens in solution can be activated by introducing a bad solvent, such as H_2_O, to induce aggregation. As can be seen in Fig. 2b, DMTPS shows negligible emission in THF, which significantly increases upon addition of H_2_O (90 vol%), with a corresponding increase in the PLQY from non-detectable to 22.2 (±7.6)% (Table S1, ESI†), clearly demonstrating its AIE behaviour. However, DMTPS-Sil is more soluble in water due to the presence of the ureido groups in the structure that can participate in hydrogen-bonding. Consequently, the AIE response of DMTPS-Sil upon addition of H_2_O (90 vol%) is less dramatic (Fig. 2c), resulting in only a modest increase in the PLQY from 3.1 (±0.1) to 8.0 (±0.3)% (Table S1, ESI†).

### PL properties through the sol–gel transition

**Fig. 3:**
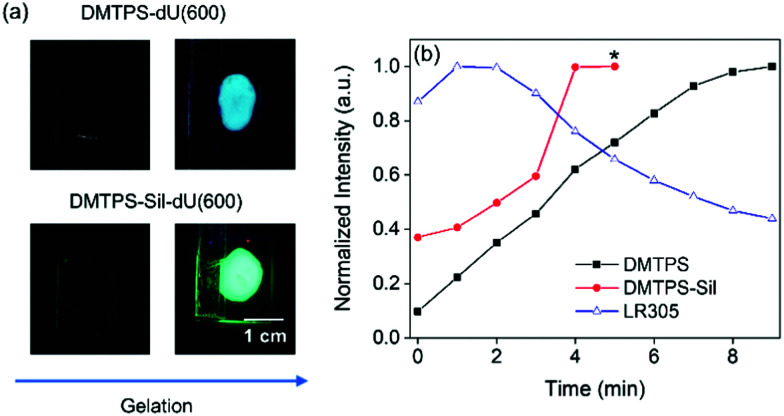
Optical properties of AIEgens during the sol–gel transition. (a) Photographs of AIEgen-d-UTPES solution mixtures immediately upon addition of the gelation reagents (left column) and the solidified AIEgen-dU(600) materials (right column) under UV irradiation (365 nm). (b) Normalised mean intensity extracted from the histograms of confocal microscopy images recorded throughout the gelation process (time interval ∼1 min) using laser excitation of 405 nm (for DMTPS-d-UPTEs/DMTPS-Sil-d-UPTES) and 488 nm (for LR305-d-UPTES). At *t* = 0 min, the gelation reagents are added to the lumophore-d-UPTES solution to initiate the sol–gel reaction. All samples had a lumophore concentration of 1.4 mM with respect to the solidified samples. *The emission saturated the detector at this point for DMTPS-Sil, preventing quantification of the intensity at longer times.

The emission properties of the AIEgens transitioning from solution to solid state through the sol–gel process were examined next. Fig. 3a shows photographs of DMTPS-dU(600)-1.4 mM and DMTPS-Sil-dU(600)-1.4 mM on a glass slide under UV irradiation (365 nm), taken before and after the gelation. These samples were chosen as representative examples of the physically doped and covalently grafted systems, respectively, in which the lumophore concentration is sufficiently high to allow for direct comparison with a lumophore (LR305) known to display aggregation-caused quenching at this concentration.

Activation of the AIE emission upon gelation of the d-UPTES precursor is visibly demonstrated in Fig. 3a, where the PL intensity of the solidified samples is significantly greater than that of the corresponding pre-gel solution states. The change in emission intensity throughout the gelation process was monitored more closely using confocal fluorescence microscopy (CFM). The samples were excited using a 405 nm laser and the resulting images are shown in Fig. S8 (ESI†). Fig. 3b shows the normalised mean-intensity change of the emission (>500 nm) as the gelation progresses, quantitatively extracted from the histograms of the CFM images, which were taken at time intervals of 1 min. At *t* = 0 min, the reagents were added to initiate the gelation process and at *t* = 9 min, the sample is fully gelled and no further noticeable change in the mean-intensity was observed. As can be seen from Fig. 3b, both DMTPS and DMTPS-Sil (*x* = 1.4 mM) showed a continuous increase in emission intensity over time, and at *t* = 5 min, the emission from DMTPS-Sil becomes too intense and saturates the detector, preventing further quantitative measurement. An analogous sample was prepared using the organic dye Lumogen Red 305 (LR305) and investigated in the same way. LR305 is a widely-adopted perylene diimide dye due to its high emission efficiency,^66,67^ but it is susceptible to ACQ at concentrations above 0.07 mM in di-ureasils.^50^ As expected, as the sol–gel transition occurs, continually increased quenching of the LR305 emission is observed (Fig. S8, ESI† and Fig. 3b). This result clearly demonstrates the role of the di-ureasil host in promoting aggregation of organic lumophores dispersed within, which can be harnessed to switch on the emission of AIEgens, but leads to ACQ of conventional lumophores such as LR305. We note that analogous experiments on samples containing lower concentrations of the AIEgen would still be expected to exhibit the AIE response (as investigated in detail in the final solid samples in the next section), but would not allow for comparison with the LR305 system as significant ACQ is not present at such low concentrations.

### Optical properties of AIEgens in solid di-ureasils

**Fig. 4:**
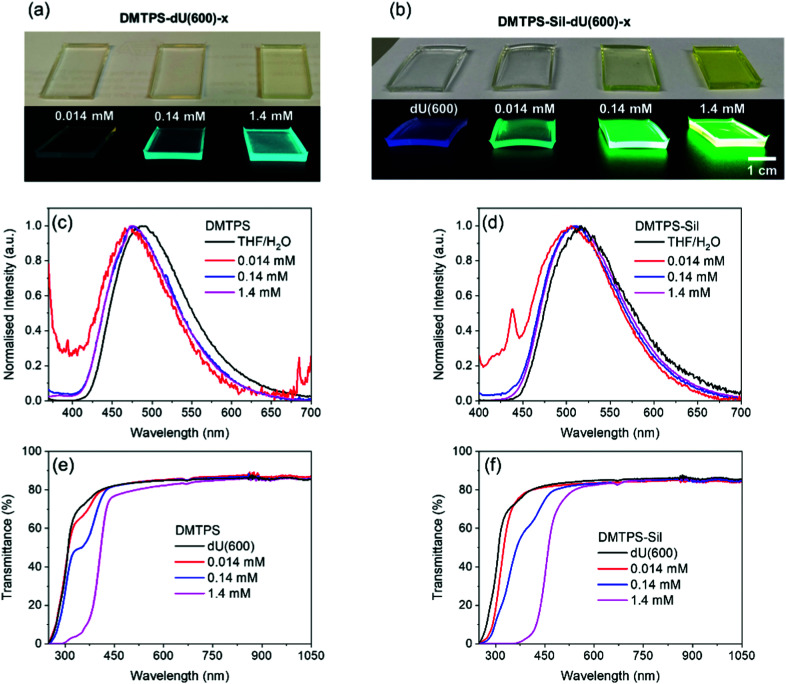
Optical properties of AIEgen-dU(600) ureasils. Photographs of (a) DMTPS-dU(600)-*x* and (b) DMTPS-Sil-dU(600)-*x* monoliths under daylight (top) and UV-irradiation (365 nm, bottom). Normalised emission spectra of (c) DMTPS (*λ*_ex_ = 370 nm) and (d) DMTPS-Sil (*λ*_ex_ = 390 nm) in a 10 : 90 (v/v) THF : H_2_O solvent mixture (solid black line) and in dU(600) at varying concentrations. UV/Vis/NIR transmittance spectra of (e) DMTPS-dU(600)-*x* and (f) DMTPS-Sil-dU(600)-*x*. Note: the distinct peaks in the short (*ca.* 440 nm) and long (*ca.* 680 nm) wavelength regions for the lowest AIEgen concentrations in (c) and (d) arise due to scattering artefacts from the sample surface. These are more prominent for these weakly emissive samples.

Photographs of the monolithic solid-state samples of DMTPS-dU(600)-*x*, DMTPS-Sil-dU(600)-*x* and the reference dU(600) are shown in Fig. 4a and b. Notably, the hybrid materials exhibit excellent transparency and homogeneity under daylight conditions and intense PL emission when irradiated using UV light (365 nm). The normalised emission spectra (Fig. 4c and d) show that the emission maxima of DMTPS-dU(600)-*x* and DMTPS-Sil-dU(600)-*x* are centred at around 470 nm and 510 nm, respectively. Negligible red-shift or distortion in the emission spectra is observed as the AIEgen concentration increases, suggesting minimal re-absorption of the emitted photons, which is expected due to the large Stokes shift (Fig. 2a).^9^ It should also be noted that the emission spectrum of DMTPS dispersed in the dU(600) matrix is blue-shifted (Δ*λ*_em_ ∼ 15 nm, Table S2, ESI†) compared to the parent solution. The same feature is not observed for DMTPS-Sil, which is covalently-grafted to the di-ureasil host. This indicates that the AIEgen might adopt different molecular conformations or intermolecular interactions when directly dispersed rather than covalently-grafted to the hybrid matrix. For example, hydrogen bonding interactions between neighbouring ureido groups have been previously observed in organic–inorganic silicates, and may lead to an enhanced PLQY.^68,69^ However, unfortunately structural characterisation of the embedded lumophores in the current system is challenging due to the low density of dye molecules present in the host matrix and the amorphous nature of the ureasil.^54^

The transmittance spectra of the samples in the UV/Vis/NIR region (250–1050 nm) are shown in Fig. 4e and f. The parent dU(600) host exhibits a high transmittance of >80% in the visible and NIR region, which is comparable to the transmittance of commonly used optically-neutral polymer hosts such as polycarbonate and PMMA.^70^ This demonstrates the excellent potential of di-ureasils as host materials in optical applications such as LSCs^50,71,72^ or visible light communication.^10,11^ As expected, both DMTPS-dU(600)-*x* and DMTPS-Sil-dU(600)-*x* show increasing absorption from the AIEgens across the UV and blue region as the concentration increases, reaching saturation in the UV region at the highest dopant concentration of 1.4 mM. There is also a slight loss of transmittance for DMTPS-dU(600)-1.4 mM (Fig. 4e) in the long-wavelength region (>600 nm) of the spectrum where neither dU(600) nor DMTPS absorb light. This indicates the presence of scattering losses in this sample caused by sufficiently large aggregates of DMTPS. However, no loss of transmittance is observed for DMTPS-Sil-dU(600)-1.4 mM suggesting that covalent grafting to the di-ureasil host improves the dispersion of the AIEgen to avoid such scattering losses.

On moving from the THF/H_2_O solvent system to the solid dU(600) matrix, the PLQY of DMTPS-Sil increases significantly from 8.0 (±0.3) to 36.7 (±2.1)% for DMTPS-Sil-dU(600)-0.014 (*λ*_ex_ = 400 nm), whilst the PLQY of DMTPS remains essentially unchanged at around 20% for DMTPS-dU(600)-0.014 (Table 1). Furthermore, the PLQY of DMTPS-Sil is always higher than that of DMTPS for the same loading content in the dU(600) matrix (Table 1). Thus, while transmittance measurements suggest the presence of larger aggregates of the AIEgen in the DMTPS-dU(600) system, this does not seem to translate to increased PLQY. In contrast, the additional ureido side groups present on the DMTPS-Sil that facilitate hydrogen bonding, coupled with covalent grafting to the ureasil matrix, appear to further restrict intramolecular rotations, leading to the observed higher PLQYs.

**Table 1:** Decay lifetimes (*τ*_*i*_), pre-exponential coefficients (*α*_*i*_) and chi-squared (*χ*^2^) values obtained from the reconvolution analysis of emission decay curves measured for the DMTPS-Sil-dU(600)-*x* and DTMPS-dU(600)-*x* series, detected at the emission wavelength of 600 nm, upon excitation at 375 nm. The PLQY values of each sample are also reported

Sample	*τ*_1_ (ns)	*τ*_2_ (ns)	*τ*_3_ (ns)	*τ*_4_ (ns)	*α*_1_	*α*_2_	*α*_3_	*α*_4_	*χ* ^2^	PLQY^a^
dU(600)	0.87	—	4.51	11.74	0.64	—	0.28	0.08	1.46	4.1% ± 0.4%
DMTPS-Sil-dU(600)-0.014	0.71	2.04	4.52	11.38	0.44	0.28	0.22	0.05	1.16	36.7% ± 2.1%
DMTPS-Sil-dU(600)-0.14	0.68	1.98	4.70	11.23	0.32	0.51	0.16	0.02	1.28	68.1% ± 6.9%
DMTPS-Sil-dU(600)-1.4	0.40	2.18	4.79	11.72	0.28	0.50	0.22	0.01	1.21	76.9% ± 4.1%
DMTPS-dU(600)-0.014	0.64	2.45	4.54	12.29	0.48	0.16	0.36	0.07	1.21	20.3% ± 4.0%
DMTPS-dU(600)-0.14	0.62	2.28	5.51	12.52	0.41	0.21	0.35	0.03	1.07	43.1% ± 1.8%
DMTPS-dU(600)-1.4	0.56	2.55	5.53	12.20	0.09	0.39	0.50	0.02	1.34	52.6% ± 2.2%

a Average values and errors are the mean and standard deviation of three independent measurements (*λ*_ex_ = 370 nm for dU(600) and 410 nm for DMTPS-Sil-dU(600)-*x* and DMTPS-dU(600)-*x*).

Dye extraction experiments were also performed to confirm the mode of interaction of the AIEgen with the di-ureasil host. A small piece of the parent sample of known mass was immersed in neat acetone, a good solvent for both AIEgens and the di-ureasil, for 72 hours. The sample was then removed, and the emission spectrum of the immersion solution was recorded (Fig. S9 and S10, ESI†). It is clear that DMTPS, which is physically dispersed into the dU(600) matrix, is leached into the acetone solution, and the acetone-soaked DMTPS-dU(600)-1.4 mM sample has lost most of its emission under UV irradiation (Fig. S9a, ESI†). In contrast, no leaching of DMTPS-Sil, which is covalently-grafted to the host, is observed from the measured emission spectrum and DMTPS-Sil-dU(600)-1.4 mM maintains its emission under UV illumination after being soaked in acetone (Fig. S9c, ESI†). These results confirm that the DMTPS-Sil molecules are chemically grafted to the di-ureasil framework, whereas the DMTPS molecules are only physically confined within the matrix.

### Time-resolved photoluminescence studies

PL lifetime measurements were performed to gain further insight into the electronic interaction between the AIEgen and the di-ureasil host for the two incorporation methods. Firstly, the emission lifetimes of the AIEgens in solution were characterised, with excitation at 375 nm and emission detected at 600 nm. This emission wavelength was chosen to minimise any contribution from the ureasil to the emission, such that the emission from the AIEgens could be captured selectively (Fig. S11, ESI†). In THF solution, the decay curve of DMTPS-Sil (Fig. S12a, ESI†) is best described by a bi-exponential function (Table S1, ESI†), with a short lifetime component, *τ*_TPS1_, attributed to the monomeric species with rapid intramolecular rotation and a longer lifetime, *τ*_TPS2_, assigned to emission from a small population of aggregates, which seems reasonable at the relatively high sample concentration used (100 μM).^73,74^ The short average lifetime (*τ*_avg_) of ∼650 ps, is in reasonable agreement with the lifetimes of silole-based AIEgens in good solvents typically reported in the literature, which range from tens to a few hundred picoseconds depending on the structure of the silole compound.^75,76^ Upon addition of 90 vol% H_2_O, *τ*_avg_ increases to ∼800 ps, which is accompanied by an increase in the corresponding pre-exponential factor of the longer lifetime component (*α*_2_), from 0.03 to 0.06 (Table S1, ESI†), confirming the formation of more aggregates. Similar observations are made for DMTPS in solution (Fig. S12b and Table S1, ESI†). Notably, the increase in value of *α*_2_ (from 0.01 to 0.41) and *τ*_avg_ (from 630 ps to 3.0 ns) upon the addition of H_2_O is much higher than for the analogous DMTPS-Sil system (Table S1, ESI†). This suggests increased aggregation for DMTPS, consistent with the previous assumption about the relatively higher solubility of DMTPS-Sil in water due to the presence of hydrogen-bonding ureido groups.

**Fig. 5:**
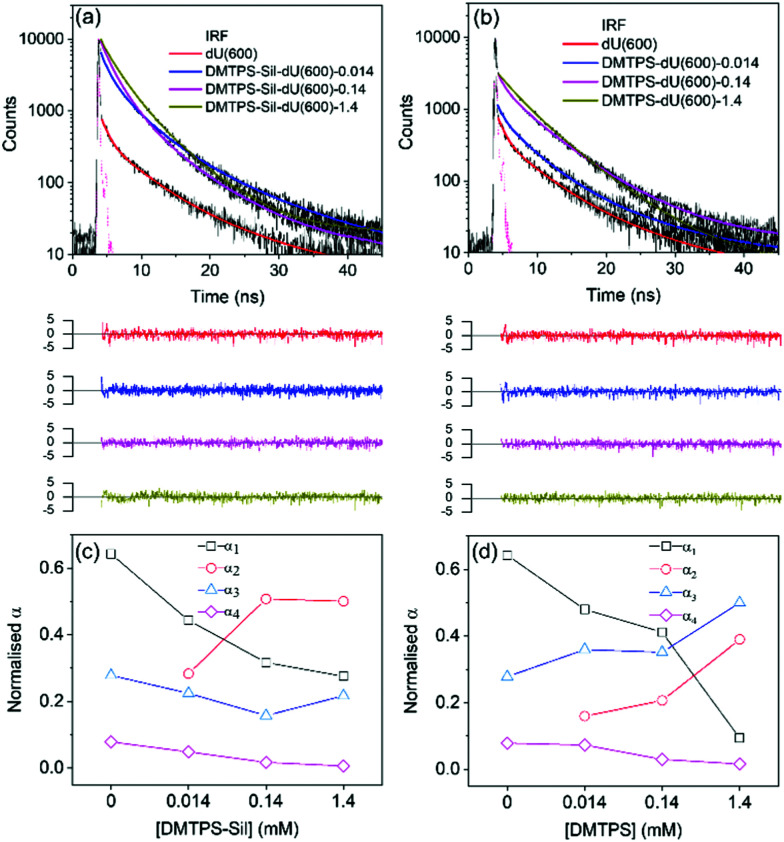
Time-resolved emission measurements on AIEgens in the di-ureasil host. Emission decay curves (solid black lines) and corresponding fits (solid coloured lines) of (a) DMTPS-Sil-dU(600)-*x* and (b) DMTPS-dU(600)-*x* upon excitation at 375 nm and detection of the emission decay curves at 600 nm. The weighted residuals for each fit and the instrument response function (IRF) (dashed pink line) are also shown. Plots of the pre-exponential factor, *α*_*i*_, associated with each lifetime component, *τ*_*i*_, against the concentration (mM) of (c) DMTPS-Sil and (d) DMTPS in the dU(600) matrix. The connecting lines serve only to guide the eye.

The emission decay curves of the AIEgens in the dU(600) matrix were considered next. As noted in the introduction, the undoped ureasil host is also intrinsically photoluminescent.^44,45^ This is clearly seen in Fig. 4b, where the reference dU(600) sample shows blue emission under UV irradiation (365 nm), which becomes masked by the more intense emission of AIEgens upon their incorporation. Upon excitation at 375 nm, the parent ureasil still displays a detectable signal at *λ*_em_ = 600 nm, which is best described as a triexponential decay curve (Fig. 5a and Table 1), in agreement with the literature.^46,54,77^ The presence of multiple lifetime components is explained by the existence of different emissive species contributing to the overall PL emission of the di-ureasil: *τ*_1_ (∼0.87 ns) and *τ*_4_ (∼11.74 ns) are attributed to the electron–hole recombination events occurring at the siliceous nanodomains through the localised oxygen defects (*τ*_DU1_) and at the urea linkages *via* proton transfer (*τ*_DU3_), respectively,^54^ whereas *τ*_3_ (∼4.51 ns) is proposed to be associated with relaxation following population by energy transfer between the two emissive species (*τ*_DU2_).^54^ After the incorporation of DMTPS-Sil, the decay curve (Fig. 5a) is best fitted with four lifetime components (Table 1). By first examining the fitting results for the sample with the lowest concentration of DMTPS-Sil (DMTPS-Sil-dU(600)-0.014), we attribute the new *τ*_2_ component (∼2.04 ns) to the PL decay of the monomeric DMTPS-Sil species (*τ*_TPS1_). The intermediate lifetime, *τ*_3_ (∼4.52 ns), is now assigned to contributions from both *τ*_DU2_ and the PL decay of the aggregated species of DMTPS-Sil in dU(600) (*τ*_TPS2_). This assignment was further confirmed by selectively exciting the DMTPS-Sil at 450 nm, while detecting the emission decay at 620 nm to avoid any PL contribution from the di-ureasil (Fig. S13 and Table S3, ESI†). We note that comparable measurements could not be obtained for DMTPS-dU(600)-*x* due to the blue-shifted absorption and emission spectra of DMTPS which overlap with the di-ureasil. It can clearly be seen that at the lowest concentration (0.014 mM), DMTPS-Sil exhibits a bi-exponential decay, with shorter (*τ*_TPS1_) and longer (*τ*_TPS2_) lifetime components at around 1.82 and 4.79 ns, corresponding to *τ*_2_ and *τ*_3_ of DMTPS-Sil-dU(600)-0.014 in Table 1, respectively. Hence, we can confidently assign the remaining two lifetimes, *τ*_1_ (∼0.71 ns) and *τ*_4_ (∼11.38 ns), entirely to the PL components of the di-ureasil host, *τ*_DU1_ and *τ*_DU3_, respectively (Table 1).

Fig. 5b shows the PL decay curves measured for DMTPS-dU(600)-*x* at the same excitation (375 nm) and emission (600 nm) wavelengths, and the corresponding fitting data are displayed in Table 1. Similar trends in the emission lifetimes and associated pre-exponential factors are observed. We note that due to the difference between the absorptivity and PLQY of DMTSP-Sil and DMTPS in the dU(600) matrix at a given excitation wavelength, direct quantitative comparisons cannot be made between the two systems. Moreover as several different PL-active species contribute to the overall sample emission, the pre-exponential factor cannot represent the absolute fractional population of the emitters associated with each lifetime component (*τ*_*i*_).^58^ Nevertheless, by comparing *α* values in the same sample system, we should be able to obtain information about the change in contribution from each emissive species associated with the corresponding *τ* to the overall PL decay as a function of the AIEgen concentration.

Fig. 5c and d show how the pre-exponential factor, *α*, associated with each lifetime component changes with doping concentration for DMTPS-Sil-dU(600)-*x* and DMTPS-dU(600)-*x*, respectively. As expected, for both systems, *α*_1_ and *α*_4_ decrease with increasing AIEgen content as the ureasil contribution (*τ*_DU1_ and *τ*_DU3_) to the overall PL decay of the sample decreases. *α*_2_, which represents the contribution from *τ*_TPS1_ (*i.e.* the monomeric species), increases with concentration as more DMTPS-Sil monomers are incorporated into the dU(600) matrix. As discussed, the intermediate lifetime, *τ*_3_, contains contributions from both *τ*_DU2_ and *τ*_TPS2_, and here we begin to see differences between the two systems. For DMTPS-Sil-dU(600)-*x*, initially the decrease in *τ*_DU2_ with increasing DMTPS-Sil concentration leads to a corresponding decrease in *α*_3_. However, as the doping concentration is increased to 1.4 mM, more aggregates of DMTPS-Sil are formed, leading to higher contribution from *τ*_TPS2_, and therefore an increase in the value of *α*_3_. In contrast, for DMTPS-dU(600)-*x*, a general increase of *α*_3_ is observed across the entire concentration series. This could suggest comparatively increased aggregation for DMTPS in the dU(600) with increasing doping concentration, leading to a greater contribution from the *τ*_TPS2_ component, thereby raising the overall value of *α*_3_, despite the simultaneous decrease in the contribution from *τ*_DU2_. This is also consistent with conclusions from the UV/Vis transmittance measurements (Fig. 4e and f), where slight scattering losses due to the formation of large molecular aggregates was detected for DMTPS-dU(600) at the highest doping concentration (1.4 mM), but not for DTMPS-Sil-dU(600) at the same concentration. The most likely explanation is that covalent grafting of the AIEgen to the dU(600) improves its spatial isolation, thereby reducing the extent of molecular aggregation.

**Fig. 6:**
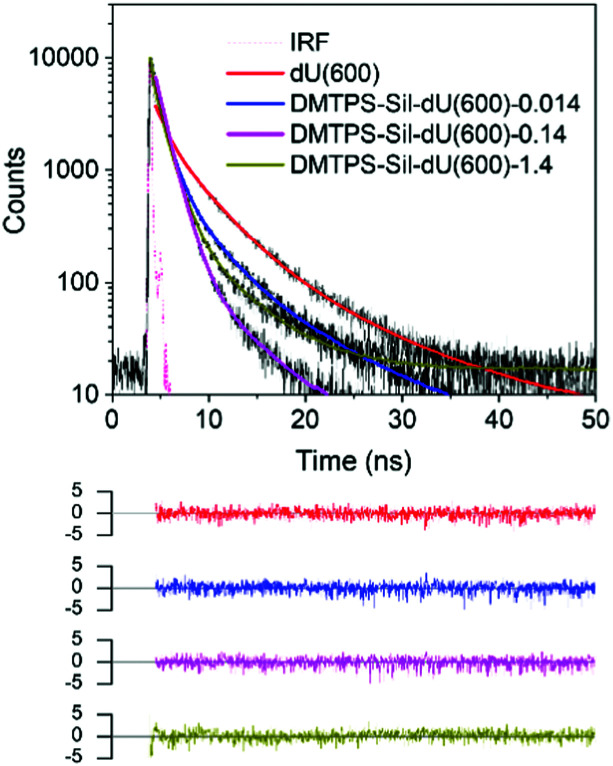
Time-resolved emission measurements provide evidence of FRET from the dU(600) host to the embedded AIEgen. Emission decay curves (solid black lines) and corresponding fits (solid coloured lines) for DMTPS-Sil-dU(600)-*x*, selectively detected in the ureasil emission band at 390 nm, upon excitation at 375 nm. The weighted residuals for each fit and the instrument response function (IRF) (dashed pink line) are also shown.

Interestingly, for both systems, a continuous decrease in *τ*_1_ with increasing AIEgen concentration was observed (Table 1). Previous studies have shown that Förster resonance energy transfer can occur from the ureasil matrix to embedded lumophores that have appropriate spectral overlap.^46,50^ Fig. S11 (ESI†) shows that there is excellent overlap between the emission spectrum of dU(600) and the absorption profile of DMTPS-Sil in solution, which provides the ideal conditions for FRET between dU(600) and DMTPS-Sil, acting as donor and acceptor, respectively. According to FRET theory, the lifetime of the donor reduces in the presence of the acceptor.^58^ Hence by selectively monitoring the emission decay of the dU(600) donor (*λ*_em_ = 390 nm) as a function of the AIEgen acceptor concentration, we can obtain valuable information about the FRET process that is potentially occurring between the two species. We note that it is not possible to identify an excitation wavelength that exclusively excites the ureasil donor; however at *λ*_ex_ = 375 nm, dU(600) is expected to be the primary absorber (Fig. S11, ESI†). The emission decay curves and corresponding fitting data are presented in Fig. 6 and Table 2, respectively. It is also worth noting that the emission intensity of dU(600) detected at 390 nm reduces significantly as the concentration of grafted DMTPS-Sil increases, which leads to increasingly prolonged data acquisition times and an elevated baseline for the decay curve for DMTPS-Sil-dU(600)-1.4 mM (Fig. 6). This suppression of the di-ureasil emission also suggests the occurrence of FRET between the two species.

**Table 2:** Decay lifetimes (*τ*_*i*_), pre-exponential coefficients (*α*_*i*_), amplitude-weighted lifetime (〈*τ*〉) and chi-squared (*χ*^2^) values obtained from the reconvolution analysis of emission decay curves measured for the DMTPS-Sil-dU(600)-*x* series containing different concentrations of DMTPS-Sil, selectively detected in the ureasil emission at 390 nm (*λ*_ex_ = 375 nm). The FRET efficiency, *E*, calculated based on the 〈*τ*〉 measured for doped and undoped samples is also presented

Sample	*τ*_1_ (ns)	*τ*_2_ (ns)	*τ*_3_ (ns)	*α*_1_	*α*_2_	*α*_3_	〈*τ*〉 (ns)	*χ* ^2^	*E* (%)
dU(600)	0.98	3.71	10.66	0.54	0.41	0.05	2.60	1.22	0
DMTPS-Sil-dU(600)-0.014	0.96	2.87	8.18	0.82	0.15	0.03	1.46	1.30	44
DMTPS-Sil-dU(600)-0.14	0.69	1.34	5.96	0.52	0.46	0.01	1.05	1.24	60
DMTPS-Sil-dU(600)-1.4	0.29	1.21	5.78	0.54	0.44	0.02	0.81	1.24	69

FRET was further confirmed by the decay curve data fits. As shown in Table 2, the lifetimes of all three decay components decrease steadily with increasing concentration of DMTPS-Sil, suggesting energy transfer occurs from all emissive centres of the ureasil matrix. This perhaps is unsurprising as the FRET efficiency is strongly dependent on the physical separation between the donor and acceptor species *via* an inverse sixth-power law.^58^ Hence as more DMTPS-Sil molecules are incorporated into the ureasil matrix, the average distance between the emissive sites of dU(600) and DMTPS-Sil decreases, leading to higher FRET efficiency and further reduction in the emission lifetime. This is confirmed by the calculated FRET efficiency values, *E*, reported in Table 2, where a maximum efficiency of 69% is observed at the highest acceptor concentration (*x* = 1.4 mM). Unfortunately, due to the blue-shifted emission of DMTPS (Fig. 4c) compared to DMTPS-Sil, its tail emission overlaps with the emission of dU(600) at 390 nm. This prevents selective detection and comparable analysis of the di-ureasil emission for the DMTPS-dU(600)-*x* series, and thus prevents direct comparison of the physical doped and chemically grafted approaches in this way. Nevertheless, the results here have provided solid evidence for the occurrence of FRET from the host material to the embedded AIEgen molecules.

## Conclusions

In summary, we have demonstrated that the inclusion of silole-based lumophores within the dU(600) di-ureasil host leads to aggregation-induced emission, the extent of which is determined by the structure of the AIEgen and the incorporation method. Physical entrapment of DMTPS in dU(600) results in a comparable AIE response to that observed in a poor solvent (90% water). In contrast, the ureido side groups on DMTPS-Sil afford good solubility in water, and thus only a modest AIE response. However, upon covalent grafting of DMTPS-Sil to the di-ureasil, a dramatic increase in the PLQY was observed as the AIE was switched-on. Both systems retain their PLQYs as the lumophore concentration is increased, contrasting with the ACQ behaviour observed for most organic lumophores. Notably, covalent grafting offers several advantages over physical entrapment of the AIEgen, including reduced scattering losses, higher PLQY and better chemical stability shown by the inability to leach from the host into an organic solvent.

In addition, emission lifetime measurements reveal excitation energy transfer from the emissive sites of the photoactive ureasil host to the covalently-attached AIEgen molecules. Depending on the requirements of the intended application, this electronic interaction between the two components could help to improve the spectral response, for example by enabling absorption of a greater portion of the UV/Visible spectrum. In summary, this study has demonstrated that chemical grafting of AIEgens to a solid-state host can deliver a pronounced AIE response, particular for lumophores that exhibit good solubility in polar solvents, which has the potential to expand the application space of these materials.

## Author contributions

GL synthesised the AIEgen-ureasil samples, performed all optical characterisation measurements and associated data analysis and drafted the initial manuscript. TJFS performed the CFM measurements and BLC assisted with the TCSPC measurements. MR synthesised the silole AIEgens. PG and SC supervised MR. PG did the TD-DFT calculations. RCE conceptualised and supervised the project, and drafted the original manuscript. All coauthors contributed to the revision and editing of the manuscript.

## Conflicts of interest

There are no conflicts to declare.

## Supplementary Material

TC-009-D1TC02794H-s001
