# Supplementary material for: Aggregation-induced emission from silole-based lumophores embedded in organic–inorganic hybrid hosts
Source: J Mater Chem C Mater. 2021 Sep 16;9(39):13914–25. doi: 10.1039/d1tc02794h (PMC8515938; doi:10.1039/d1tc02794h)
Supplement: TC-009-D1TC02794H-s001 [file TC-009-D1TC02794H-s001.pdf]

Electronic Supplementary Material (ESI) for Journal of Materials Chemistry C.
This journal is © The Royal Society of Chemistry 2021

Electronic Supporting Information

Aggregation-Induced Emission from Silole-Based Lumophores Embedded in Organic-Inorganic Hybrid Hosts

Guanpeng Lyu,[a] Thomas J. F. Southern,[a] Bethan L. Charles,[a] Maxime Roger,[b] Philippe Gerbier,[b] Sébastien Clément[b] and Rachel C. Evans[a]*

[a] Department of Material Science and Metallurgy, University of Cambridge, 27 Charles Babbage Road, Cambridge CB3 0FS, United Kingdom.

[b]Institut Charles Gerhardt de Montpellier (ICGM, UMR 5253), Université de Montpellier, CNRS, ENSCM, Montpellier, France.

*Corresponding author: rce26@cam.ac.uk

Table of Contents

1. Characterisation data for the silylated DMTPS derivative 3

2. ATR-FTIR spectrum for ureasil synthesis 5

3. Time-dependent density functional theory calculations 6

4. Confocal fluorescence microscopy (CFM) during the gelation process 7

5. Normalised emission spectra of DMTPS-dU(600) and DMTPS-Sil-dU(600) 8

6. Raman scattering of acetone 9

7. Photoluminescence Spectra of dU(600) and DMTPS-Sil 9

8. Emission decay of DMTPS and DMTPS-Sil in THF/H_2O solution 10

9. Emission maxima of DMTPS and DMTPS-Sil in solution and dU(600) matrix 11

10. Data fitting procedure for fluorescence decay curves 12

11. Emission decay of DMTPS-Sil in the dU(600) matrix 14

12. References 15

1. Characterisation data for the silylated DMTPS derivative

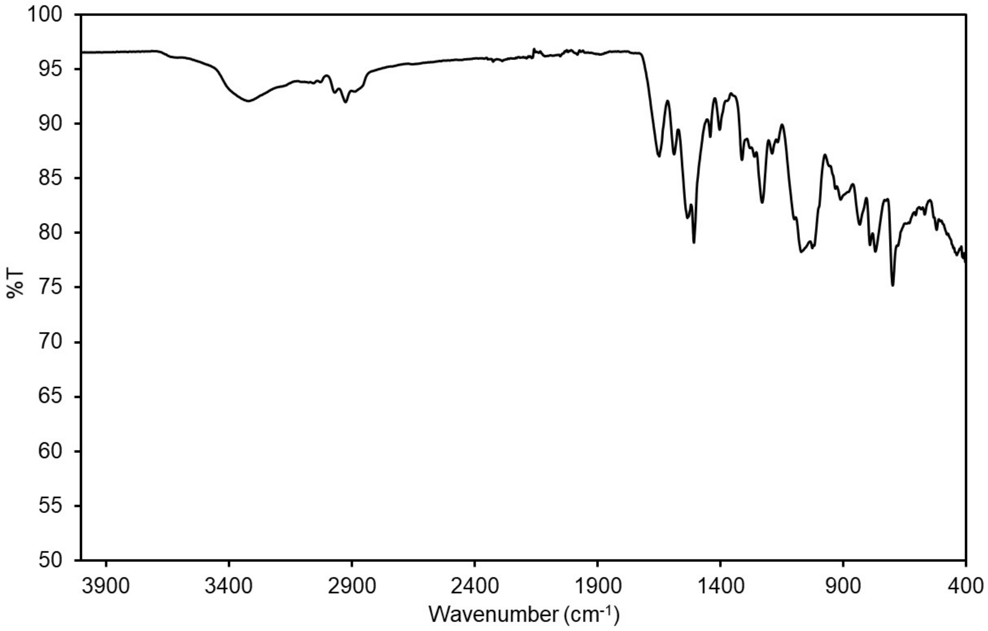

Figure S1. Attentuated total reflectance Fourier transform infrared (ATR-FTIR) spectrum of DMTPS-Sil.

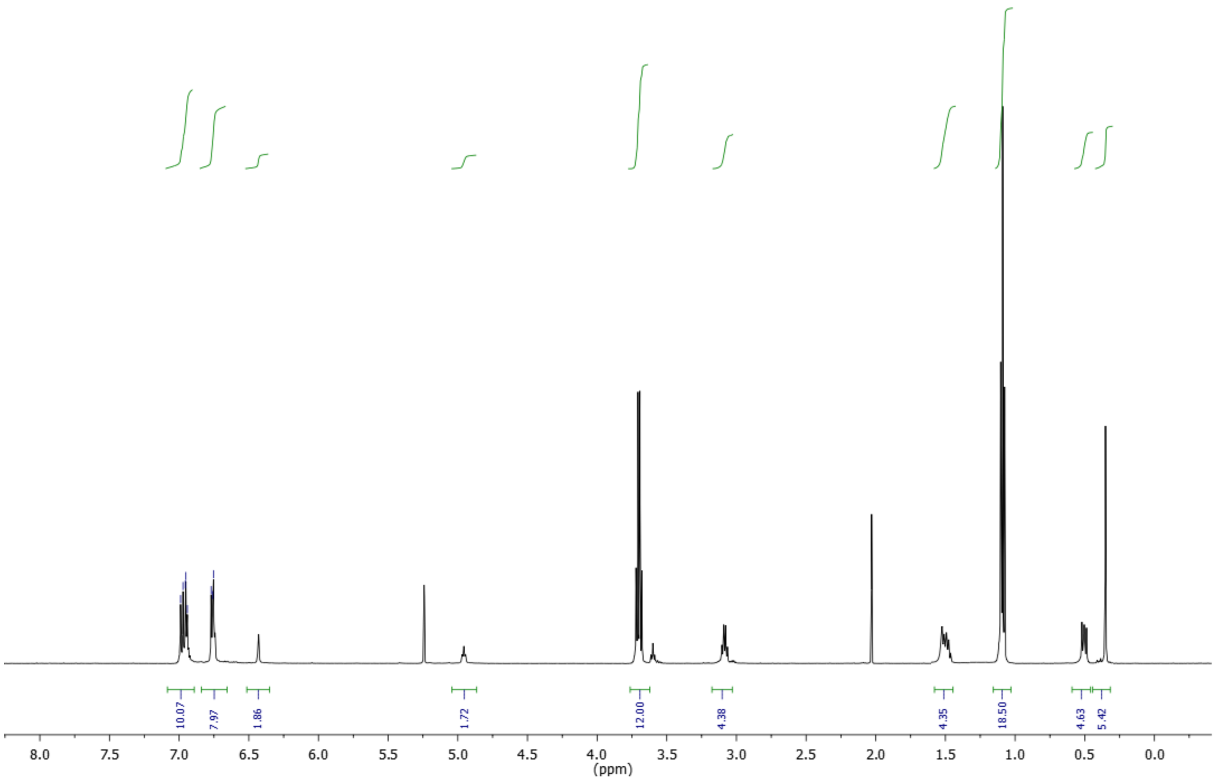

Figure S2. ¹H nuclear magnetic resonance (NMR) spectrum of DMTPS-Sil in CD_2Cl_2 at 293 K.

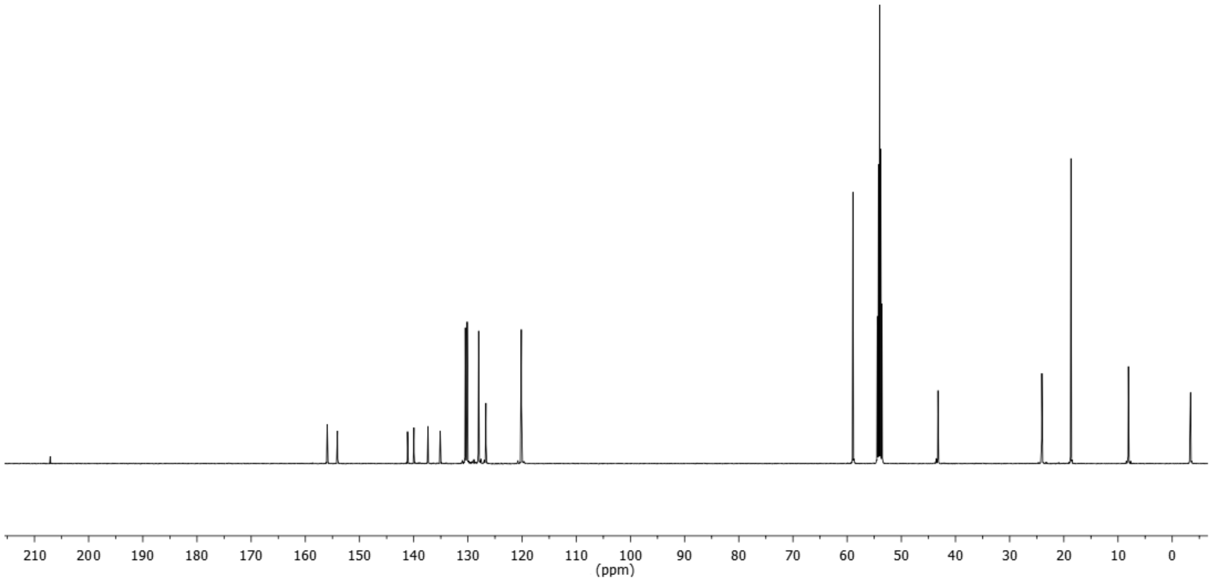

Figure S3. $^{13}C\{^{1}H\}$ NMR spectrum of DMTPS-Sil in CD_2Cl_2 at 293 K.

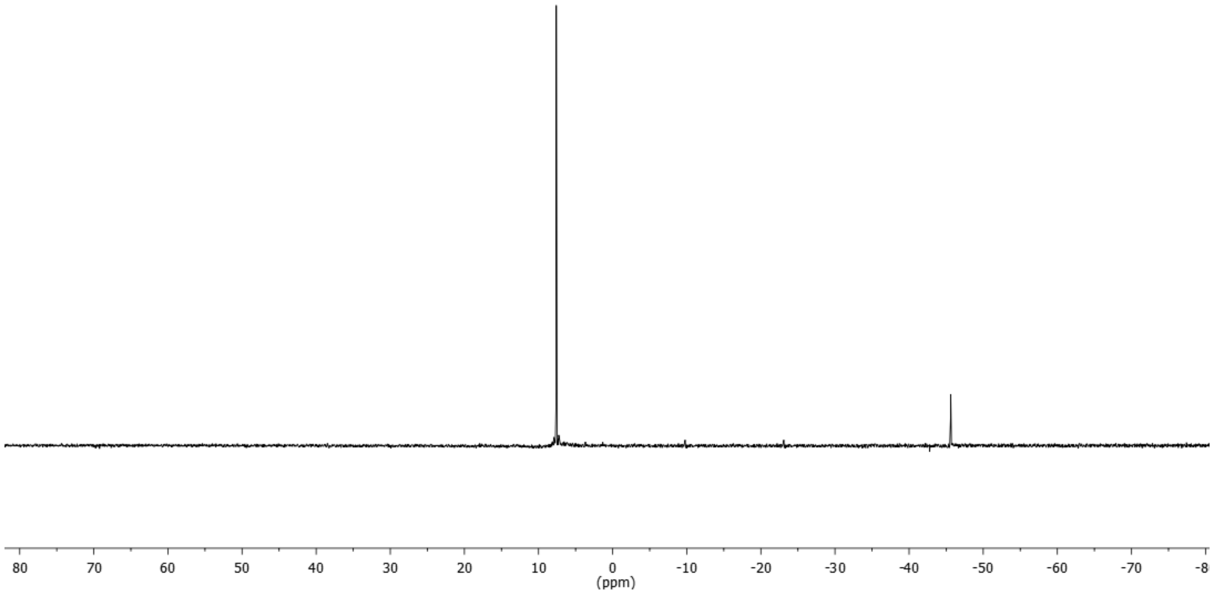

Figure S4. $^{29}Si\{^{1}H\}$ NMR spectrum of DMTPS-Sil in CD_2Cl_2 at 293 K.

2. ATR-FTIR spectrum for ureasil synthesis

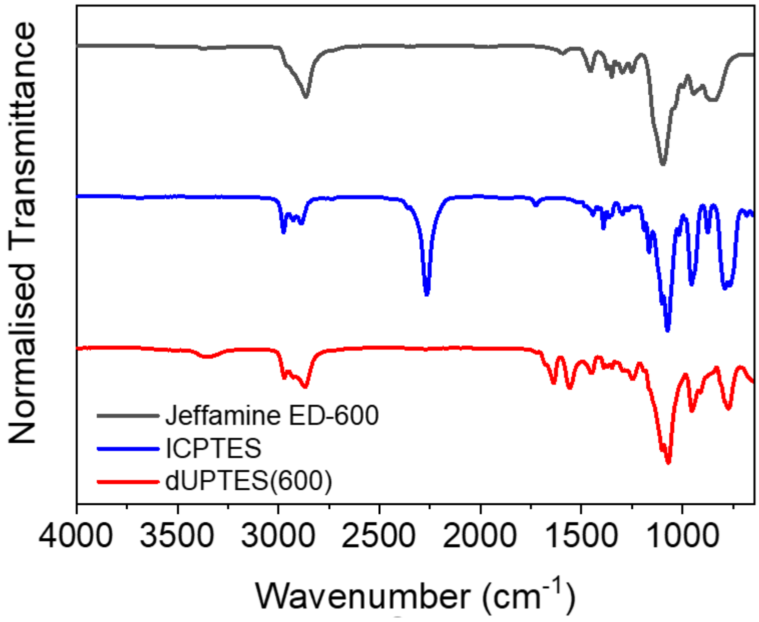

Figure S5. FTIR spectrum of the precursor Jeffamine ED-600, ICPTES and their product d-UPTES. Several distinctive features in the FTIR spectrum of dUPTES indicate the completion of the reaction between Jeffamine ED-600 and ICPTES:[1] (1) The disappearance of isocyanate stretching centred at around 2270 cm^{-1}. (2) The formation of a new band centred at around 3350 cm^{-1}, which represents the N-H stretch of the newly-formed urea linkages. (3) Two new bands appeared at around 1635 and 1550 cm^{-1}, corresponding to the C=O stretching and N-H bending of the newly-formed urea groups, respectively.

3. Time-dependent density functional theory calculations

Full geometry optimization of the ground state and frequency calculations were performed with Density Functional Theory (DFT)[2] using the hybrid Becke parameters exchange functional and the Lee-Yang-Parr non-local correlation functional (B3LYP) implemented in the Gaussian 09 program suite[3] using the 6-31+G(d,p) basis set and the default convergence criterion implemented in the program. Transition diagrams were obtained through TD-DFT calculations performed using the B3LYP functionals and the 6-311+G(d,p) basis set on the geometry of S_0.

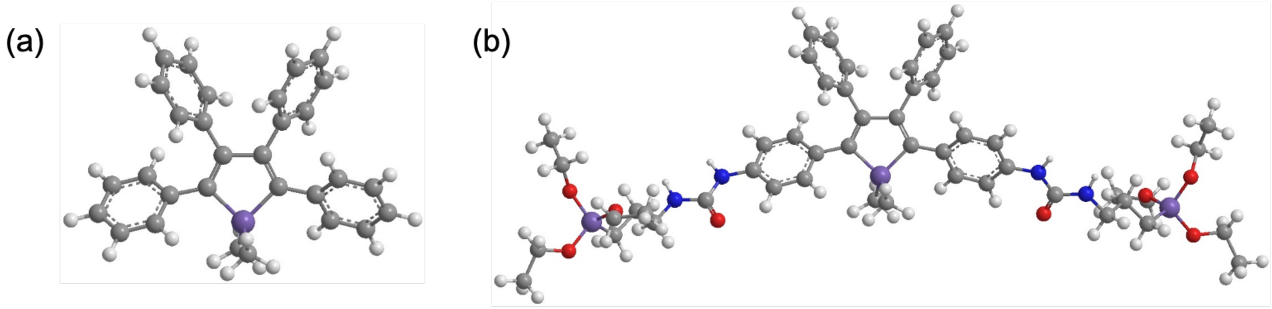

Figure S6. Optimised structures of (a) DMTPS and (b) DMTPS-Sil. Atom labels: carbon (grey), hydrogen (white), silicon (purple), nitrogen (blue) and oxygen (red). Based on the optimised structures, the torsion angles calculated between silole and phenyl groups at the 2 and 5-positions are lower in DMTPS-Sil (42.9°) than those in DMTPS (46.7°) indicating improved conjugation in DMTPS-Sil. The phenyl rings at the 3- and 4-positions are clearly unaffected by the presence of the lateral chains in the DMTPS-Sil structure.

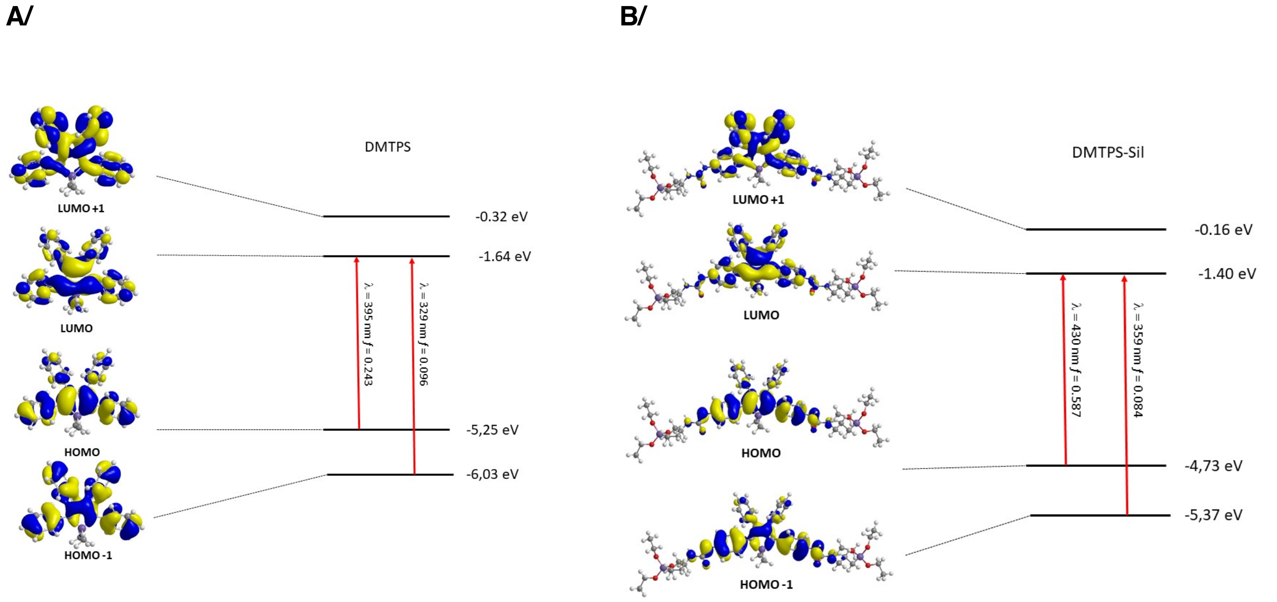

Figure S7. Representation of the energy levels and the main molecular orbitals involved in the electronic transitions of A/ DMTPS and B/ DMTPS-Sil obtained by TD-DFT B3LYP and the 6-311+G(d,p) basis set on the geometry of S_0.

4. Confocal fluorescence microscopy (CFM) during the gelation process

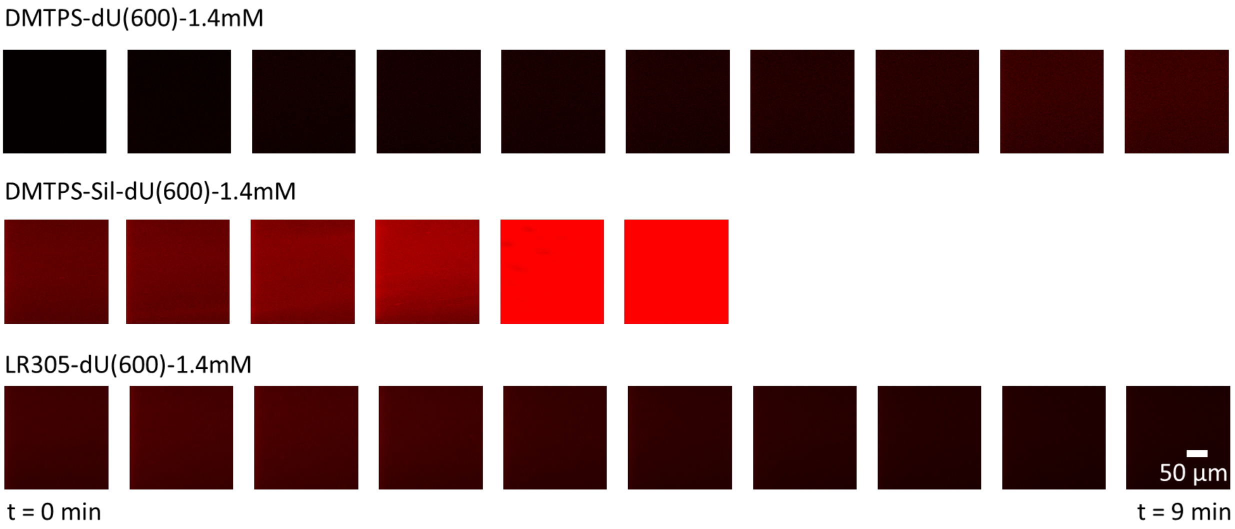

Figure S8. CFM images recorded throughout the gelation process using laser excitations of 405 nm (for DMTPS-dUPTES and DMTPS-Sil-dUPTES mixtures) and 488 nm (for LR305-dUPTES mixture), all samples with doping concentration of 1.4 mM. At time = 0 min, the gelling reagents are added to the lumophore-dUPTES solution mixture to initiate the gelation process.

5. Normalised emission spectra of DMTPS-dU(600) and DMTPS-Sil-dU(600)

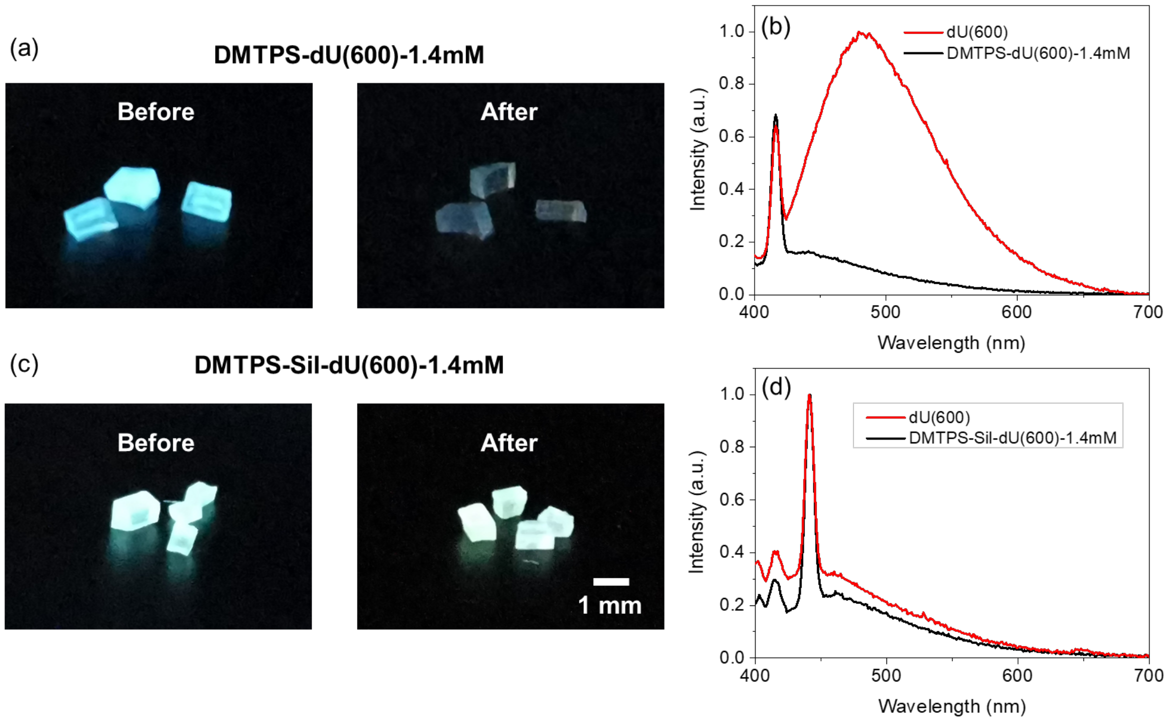

Figure S9. Photographs of small segments of (a) 1.4 mM DMTPS-dU(600) and (c) 1.4 mM DMTPS-Sil-dU(600) under UV irradiation (365 nm) before and after immersed in acetone for 72 hours. Emission spectra of the filtered acetone solution used to immerse (b) DMTPS-dU(600) and dU(600) (λex = 370 nm) and (d) DMTPS-Sil-dU(600) and dU(600) (λ_{ex} = 390 nm). The weak and broad emission centred at around 450 nm, present in the emission spectra of both acetone solutions that are used to soak the DMTPS-Sil-dU(600) and blank dU(600), is attributed to the unreacted Jeffamine and/or dUPTES precursors that are dissolved into the solution.[4] In addition, the sharp peaks located at around 410 and 440 nm are Raman scattering of liquid acetone when excited at 370 and 390 nm, respectively.[5] This is explained in more detail in Figure S10 below.

6. Raman scattering of acetone

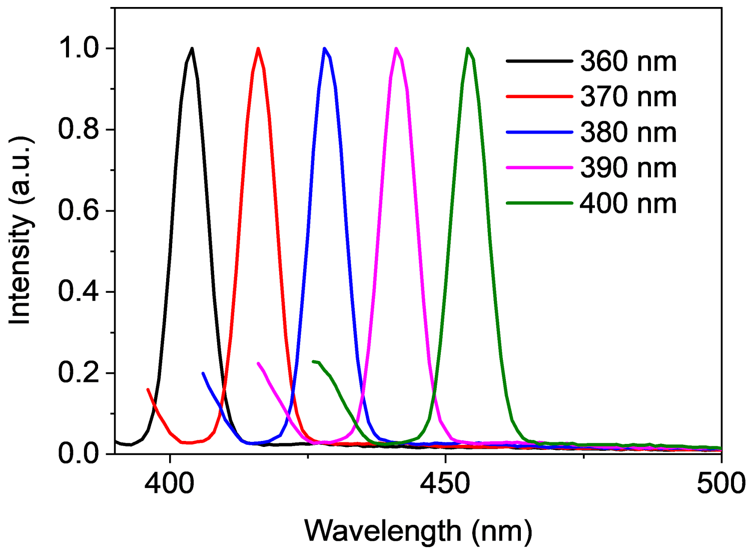

Figure S10. Normalised emission spectra of acetone as a function of excitation wavelength under ambient conditions. The difference (in wavenumbers) between the spectral position of the excitation source and centre of the peak in the emission spectrum is a constant value of 2965 cm^{-1}, which matches the IR peak position for the v_{16} (A') vibrational mode of liquid acetone,[5] demonstrating that the sharp peaks located at around 410 and 440 nm in Figure S9b and 9d, respectively, are due to Raman scattering of the liquid acetone.

7. Photoluminescence Spectra of dU(600) and DMTPS-Sil

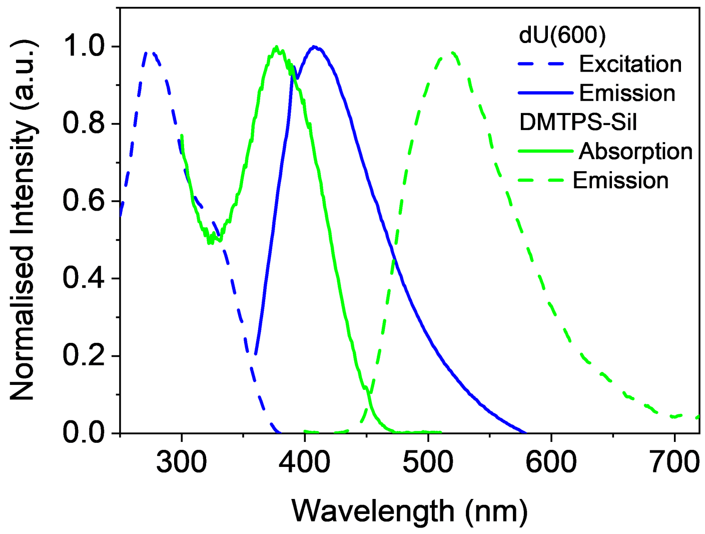

Figure S11. Normalised excitation (dashed blue, λ_{em} = 390 nm) and emission (solid blue, λ_{ex} = 350 nm) spectra of dU(600) and normalised absorption (solid green) and emission (dashed green, λ_{ex} = 390 nm) spectra of DMTPS-Sil measured in a 10:90 (by volume) THF:H2O solvent mixture at a concentration of 20 μM.

8. Emission decay of DMTPS and DMTPS-Sil in THF/H_2O solution

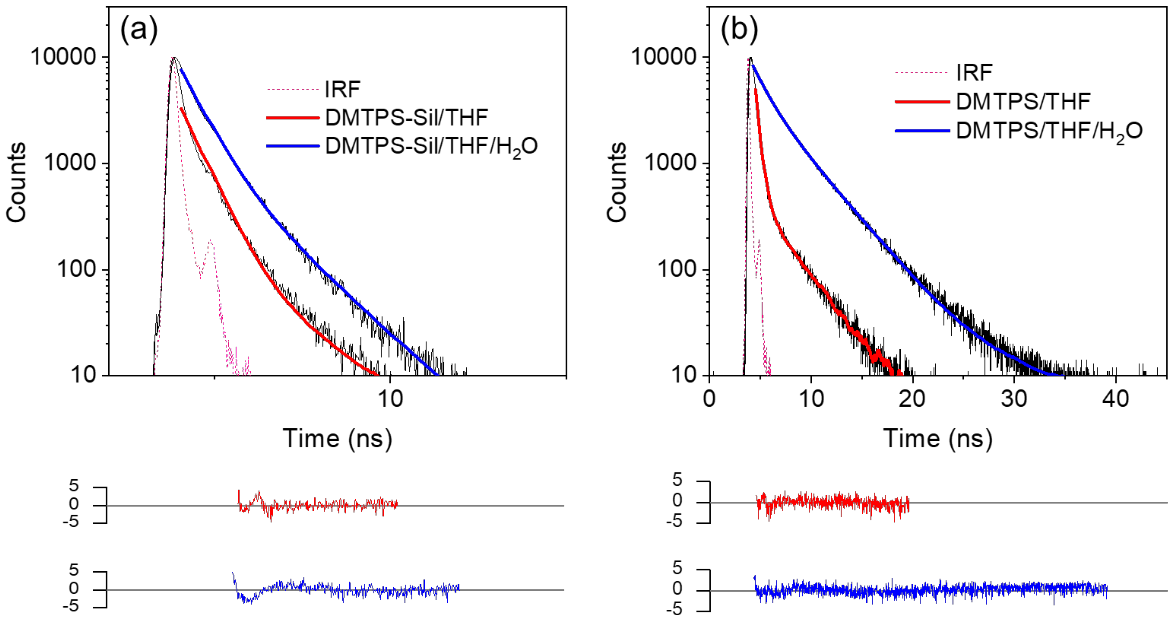

Figure S12. Time-resolved emission measurements on AIEgens in solution. Emission decay curves (solid black lines) and corresponding fits (solid coloured lines) of (a) DMTPS-Sil and (b) DMTPS in THF and 10:90 (*v/v*) THF:H_2O solvent mixture, upon excitation at 375 nm and detection of the emission decay curves at 600 nm. The weighted residuals for each fit and the instrument response function (IRF) (dashed pink line) are also shown.

Table S1. Decay lifetimes (τ_i), pre-exponential coefficients (α_i), average lifetime (τ_{avg}) and chi-squared (χ^2) resulting from the reconvolution analysis of emission decays of DMTPS-Sil and DMTPS in THF and 10:90 (*v/v*) THF:H_2O solvent mixture, detected at the emission wavelength of 600 nm, upon excitation at 375 nm. The PLQY values of each sample are also reported in the table.

Sample	τ_1	τ_2	α_1	α_2	τ_{avg}	χ^2	PLQY[a]
DMTPS-Sil/THF	0.52	1.92	0.97	0.03	0.65	1.22	3.1% ± 0.1%
DMTPS-Sil/THF/H_2O	0.54	1.93	0.94	0.06	0.80	1.16	8.0% ± 0.3%
DMTPS/THF	0.32	3.45	0.99	0.01	0.63	1.39	-[b]
DMTPS/THF/H_2O	1.35	3.84	0.59	0.41	3.00	1.22	22.2% ± 7.6%

[a]Average values and errors are the mean and standard deviation of three independent measurements (λ_{ex} = 400 nm). [b] Emission not detectable.

9. Emission maxima of DMTPS and DMTPS-Sil in solution and dU(600) matrix

Sample	DMTPS	DMTPS-Sil
	λ_{max} (nm)	λ_{max} (nm)
20 uM THF	-	520
20 uM THF/H_2O	487	517
0.014 mM dU(600)	475	505
0.14 mM dU(600)	475	509
1.4 mM dU(600)	474	509

Table S2. Emission maxima (λ_{max}) of DMTPS and DMTPS-Sil measure in THF, 10:90 (by volume) THF:H_2O solvent mixture and in dU(600) at varying concentrations. The excitation wavelength used to measure the emission spectra of DMTPS and DMTPS-Sil were 370 nm and 390 nm, respectively.

10. Data fitting procedure for fluorescence decay curves

Fluorescence decays were measured for blank and DMTPS-Sil-doped dU(600) ureasils, upon excitation at 375 and 420 nm to selectively detect the emission from dU(600) (390 nm) and DMTPS-Sil (650 nm), respectively. All fluorescence decays detected show complex multi-exponential behaviour, with two or three exponential components required to fit the data. In theory, a multi-exponential decay function can be described by the following equation:

$$I(t) = \sum_i \alpha_i \exp\left(-\frac{t}{\tau_i}\right) \tag{S1}$$

where $I(t)$ is the theoretical emission intensity at any given time t, α_i and τ_i are the pre-exponential factor and the characteristic lifetime for component I, respectively. This equation models the response of a sample to an infinitely sharp excitation (δ-function) and α_i represents the fraction of molecules with each decay component at $t = 0$.[6]

The fractional contribution f_i of each component to the overall steady-state intensity of the fluorescence emission can be calculated based on formula below:

$$f_i = \frac{\alpha_i \tau_i^2}{\sum \alpha_i \tau_i^2} \tag{S2}$$

The average emission lifetime $<\tau>$ can be calculated from:

$$< \tau > = \frac{\sum \alpha_i \tau_i^2}{\sum \alpha_i \tau_i} \tag{S3}$$

However, the excitation pulse is never an infinitely sharp δ-function in reality and the instrument response function (IRF), including the width of the excitation pulse and various electronic responses of the instrument, must be taken into account. Therefore, the theoretical emission decay ($I(t)$) of the sample is reconvoluted with the IRF in the form below:

$$I'(t) = \int_0^t E(t')I(t-t')dt' \tag{S4}$$

where $E(t)$ is the excitation pulse and $I'(t)$ is the experimental emission intensity at any given time t. This equation describes that $I'(t)$ is calculated from the sum of intensities expected for all δ-function excitation pulses that occur until time t, where the excitation pulse is comprised of a series of functions with different amplitudes.[6]

Non-linear least square analysis was then used to fit the measured emission decay based on the model described above. α_i and τ_i were varied until χ^2, the goodness-of-fit parameter, is at minimum, a method known as iterative deconvolution:

$$\chi^2 = \sum_{i=1}^{n} [\frac{y_i - f_{ic}}{y_i^2}]^2 \quad \text{(S5)}$$

where n is the number of data points, y_i is the experimental data and f_{ic} is the calculated fit. The quality of the fitted curves was also qualitatively judged by the randomness of the residuals, which is measured based on the vertical deviation of the experimental data from the fitted curves. Non-randomness in the plot of residuals suggest a poor fit or hidden variables.

11. Emission decay of DMTPS-Sil in the dU(600) matrix

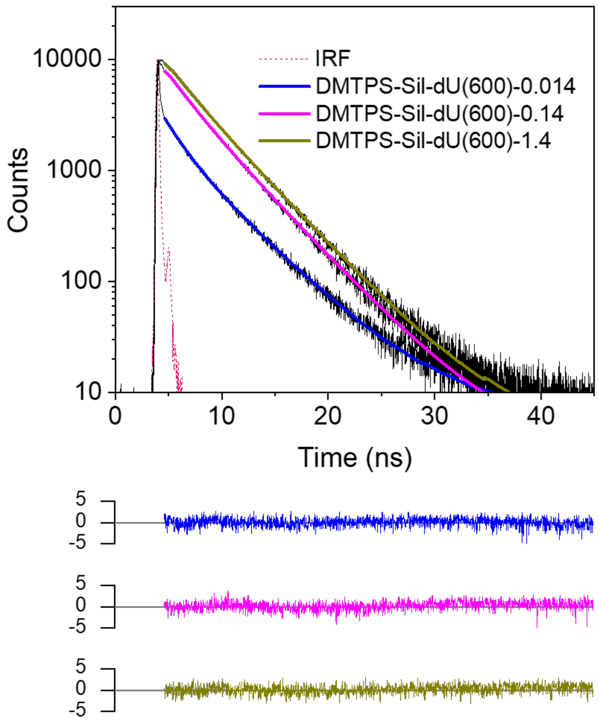

Figure S13. Emission decay curves (solid black lines) and corresponding fits (solid coloured lines) of DMTPS-Sil-dU(600)-x with varying doping concentrations, selectively detected for the emission of DMTPS-Sil at 620 nm in the dU(600) matrix, upon excitation at 450 nm. The weighted residuals for each fit and the instrument response function (IRF) (dashed pink line) are also shown.

Table S3. Decay lifetimes (τ_i), pre-exponential coefficients (α_i) and chi-squared (χ^2) obtained resulting from the reconvolution analysis of emission decay curves measured for the DMTPS-Sil-dU(600)-*x* and DTMPS-dU(600)-x series with varying doping concentrations, detected at the emission wavelength of 600 nm, upon excitation at 375 nm. The PLQY values of each sample are also reported in the table.

Sample	τ_1 (ns)	τ_2 (ns)	α_1	α_2	τ_{avg} (ns)	χ^2	PLQY[a]
DMTPS-Sil-dU(600)-0.014	1.82	4.79	0.62	0.38	3.66	1.29	36.7% ± 2.1%
DMTPS-Sil-dU(600)-0.14	2.19	4.40	0.38	0.62	3.88	1.19	68.1% ± 6.9%
DMTPS-Sil-dU(600)-1.4	2.28	4.38	0.29	0.71	4.00	1.16	76.9% ± 4.1%

[a]Average values and errors are the mean and standard deviation of three independent measurements (λ_{ex} = 400 nm).

12. References

(1) Cui, Y.; Wang, M.; Chen, L.; Qian, G. Synthesis and Spectroscopic Characterization of an Alkoxysilane Dye Containing C. I. Disperse Red 1. *Dye. Pigment.* **2004**, *62*, 43–47.

(2) Lee, C.; Yang, W.; Parr, R. G. Development of the Colic-Salvetti Correlation-Energy Formula into a Functional of the Electron Density Formula. *Phys. Rev. B* **1988**, *37*, 785–798.

(3) Frisch, M. J.; Trucks, G. W.; Schlegel, H. B.; Scuseria, G. E.; Robb, M. A.; Cheeseman, J. R.; Scalmani, G.; Barone, V.; Mennucci, B.; Petersson, G. A.; Nakatsuji, H.; Caricato, M.; Li, X.; Hratchian, H. P.; Izmaylov, A. F.; Bloino, J.; Zheng, G.; Sonnenberg, J. L.; Hada, M.; Ehara, M.; Toyota, K.; Fukuda, R.; Hasegawa, J.; Ishida, M.; Nakajima, T.; Honda, Y.; Kitao, O.; Nakai, H.; Vreven, T.; Montgomery Jr., J. A.; Peralta, J. E.; Ogliaro, F.; Bearpark, M.; Heyd, J. J.; Brothers, E.; Kudin, K. N.; Staroverov, V. N.; Kobayashi, R.; Normand, J.; Raghavachari, K.; Rendell, A.; Burant, J. C.; Iyengar, S. S.; Tomasi, J.; Cossi, M.; Rega, N.; Millam, J. M.; Klene, M.; Knox, J. E.; Cross, J. B.; Bakken, V.; Adamo, C.; Jaramillo, J.; Gomperts, R.; Stratmann, R. E.; Yazyev, O.; Austin, A. J.; Cammi, R.; Pomelli, C.; Ochterski, J. W.; Martin, R. L.; Morokuma, K.; Zakrzewski, V. G.; Voth, G. A.; Salvador, P.; Dannenberg, J. J.; Dapprich, S.; Daniels, A. D.; Farkas, O.; Foresman, J. B.; Ortiz, J. V.; Cioslowski, J.; Fox, D. J. Gaussian 09, Revision A.02. *Gaussian Inc Wallingford CT* **2009**, *34*, Wallingford CT.

(4) Carlos, L. D.; Sá Ferreira, R. A.; Pereira, R. N.; Assunção, M.; De Zea Bermudez, V. White-Light Emission of Amine-Functionalized Organic/Inorganic Hybrids: Emitting Centers and Recombination Mechanisms. *J. Phys. Chem. B* **2004**, *108*, 14924–14932.

(5) Dellepiane, G.; Overend, J. Vibrational Spectra and Assignment of Acetone, Aαα Acetone-D3 and Acetone-D6. *Spectrochim. Acta* **1966**, *22*, 593–614.

(6) Lakowicz, J. R. Principles of Fluorescence Spectroscopy. *Springer* **2006**, *Third Edit.*
